# Potent pro-inflammatory and pro-fibrotic molecules, osteopontin and galectin-3, are not major disease modulators of laminin α2 chain-deficient muscular dystrophy

**DOI:** 10.1038/srep44059

**Published:** 2017-03-10

**Authors:** Kinga I. Gawlik, Johan Holmberg, Martina Svensson, Mikaela Einerborg, Bernardo M. S. Oliveira, Tomas Deierborg, Madeleine Durbeej

**Affiliations:** 1 Department of Experimental Medical Science, Muscle Biology Unit, Lund University, Sweden; 2 Department of Experimental Medical Science, Experimental Neuroinflammation Laboratory, Lund University, Sweden

## Abstract

A large number of human diseases are caused by chronic tissue injury with fibrosis potentially leading to organ failure. There is a need for more effective anti-fibrotic therapies. Congenital muscular dystrophy type 1A (MDC1A) is a devastating form of muscular dystrophy caused by laminin α2 chain-deficiency. It is characterized with early inflammation and build-up of fibrotic lesions, both in patients and MDC1A mouse models (e.g. *dy*^*3K*^/*dy*^*3K*^). Despite the enormous impact of inflammation on tissue remodelling in disease, the inflammatory response in MDC1A has been poorly described. Consequently, a comprehensive understanding of secondary mechanisms (impaired regeneration, enhanced fibrosis) leading to deterioration of muscle phenotype in MDC1A is missing. We have monitored inflammatory processes in *dy*^*3K*^/*dy*^*3K*^ muscle and created mice deficient in laminin α2 chain and osteopontin or galectin-3, two pro-inflammatory and pro-fibrotic molecules drastically increased in dystrophic muscle. Surprisingly, deletion of osteopontin worsened the phenotype of *dy*^*3K*^/*dy*^*3K*^ mice and loss of galectin-3 did not reduce muscle pathology. Our results indicate that osteopontin could even be a beneficial immunomodulator in MDC1A. This knowledge is essential for the design of future therapeutic interventions for muscular dystrophies that aim at targeting inflammation, especially that osteopontin inhibition has been suggested for Duchenne muscular dystrophy therapy.

Inflammation is a powerful regulator of both physiological and pathological processes in tissues. Inflammation and fibrosis trigger loss of muscle function in various types of muscular dystrophy. Congenital muscular dystrophy caused by mutations in the laminin α2 chain gene (MDC1A) is one of the most devastating forms of muscular dystrophy (both in humans and mice)^1^. Clinical symptoms include severe muscle wasting, progressive muscle weakness, joint contractures, respiratory and feeding difficulties and numerous complications. Most patients lose ambulation in childhood, suffer enormous discomfort and have dramatically decreased life-span^1^. The pathology of laminin α2 chain-deficient muscle is presented with muscle fibre degeneration/regeneration, apoptosis, acute inflammation and subsequent infiltration of connective tissue^2^,^3^,^4^,^5^,^6^,^7^,^8^,^9^,^10^,^11^,^12^,^13^. Despite the tremendous impact of inflammation on tissue remodelling in disease, the inflammatory response in MDC1A has been poorly characterized. Consequently, full understanding of secondary mechanisms (e.g. impaired regeneration, fibrosis) leading to deterioration of muscle phenotype in MDC1A is missing. Several mouse models for the disease exist, among which *dy*^*3K*^/*dy*^*3K*^ mice display complete deficiency of laminin α2 chain and adequately mirror the severe phenotype of MDC1A patients^2^.

Osteopontin is a multifunctional protein, expressed by a variety of cell types in multiple tissues^14^,^15^,^16^,^17^,^18^. It plays a major role in several fibrotic disorders^19^,^20^,^21^,^22^. Importantly, in recent studies the molecule has been attributed the status of a pro-inflammatory cytokine, as it powerfully regulates immune cell activity and fate^23^,^24^,^25^,^26^,^27^,^28^,^29^. Although osteopontin levels in normal skeletal muscle are very low^30^,^31^, pleiotropic roles of the cytokine in injured or diseased muscle have recently become evident. In injured muscle inflammatory cells and myoblasts produce osteopontin^32^ and its upregulation contributes to both muscle repair and fibrosis^30^,^31^,^32^,^33^,^34^,^35^. The complexity of osteopontin interactions is illustrated by its multidirectional influence on cells that contribute to muscle repair and/or muscle deterioration: the molecule is associated with intricate regulation of inflammation that prompts myogenic cell (myoblast) proliferation and differentiation as well as fibrogenic cell (myofibroblast) differentiation^22^,^32^,^33^,^35^. Yet, the mechanisms of osteopontin-steered inflammatory events that impact muscle phenotype have not been fully understood. Notably, the protein has been shown to be upregulated in muscles from Duchenne muscular dystrophy patients and in dystrophin-deficient *mdx* mice^31^,^34^, and has been suggested to mediate the progression of dystrophin-deficiency^31^,^36^. Consequently, the deletion of osteopontin in *mdx* mice resulted in reduced fibrosis and improvement of muscle strength, possibly through skewing the macrophage population towards a pro-regenerative phenotype, demonstrating osteopontin’s powerful properties to control macrophage polarization in the dystrophic muscle^37^. Collectively, these data suggest that inflammation is the link between myogenesis and fibrosis and osteopontin could be the immunomodulator of muscle diseases. It has even been proposed that osteopontin may be a promising therapeutic target for reducing inflammation and fibrosis in Duchenne muscular dystrophy individuals^34^. Yet, its impact on disease progress in MDC1A has not been demonstrated, although there is a dramatic increase of osteopontin expression in muscle from patients and *dy*^*w*^/*dy*^*w*^ mice (animals that express low amounts of truncated laminin α2 chain)^31^,^38^.

Galectin-3, a multifunctional β-galactoside-binding animal lectin, is also an important modulator of both acute and chronic inflammation^39^,^40^,^41^. The precise inflammatory role of galectin-3 seems to depend on the type of stimulus and organ damage. However, a majority of studies suggest galectin-3 to be pro-inflammatory during acute tissue injury^42^ whereas chronic tissue damage and inflammation lead to a shift of galectin-3 function towards wound healing, promoting formation of fibrotic tissue^43^. Galectin-3 is increased in a number of different fibrotic conditions including muscular dystrophy^44^. Galectin-3 inhibitors protect against fibrotic disorders^45^,^46^ and are currently tested for the treatment of idiopathic pulmonary fibrosis^47^. The role of galectin-3 in MDC1A has never been investigated.

Hence, in the current study we address in detail the role of pro-inflammatory molecules osteopontin and galectin-3 in MDC1A by generating laminin α2 chain-osteopontin and laminin α2 chain-galectin-3 double knockout mice (*dy*^*3K*^/*OPN* and *dy*^*3K*^/*GAL*).

## Results

### Cytokine profile in *dy* ^*3K*^/*dy* ^*3K*^ dystrophic muscle over the course of the disease

**Figure 1:**
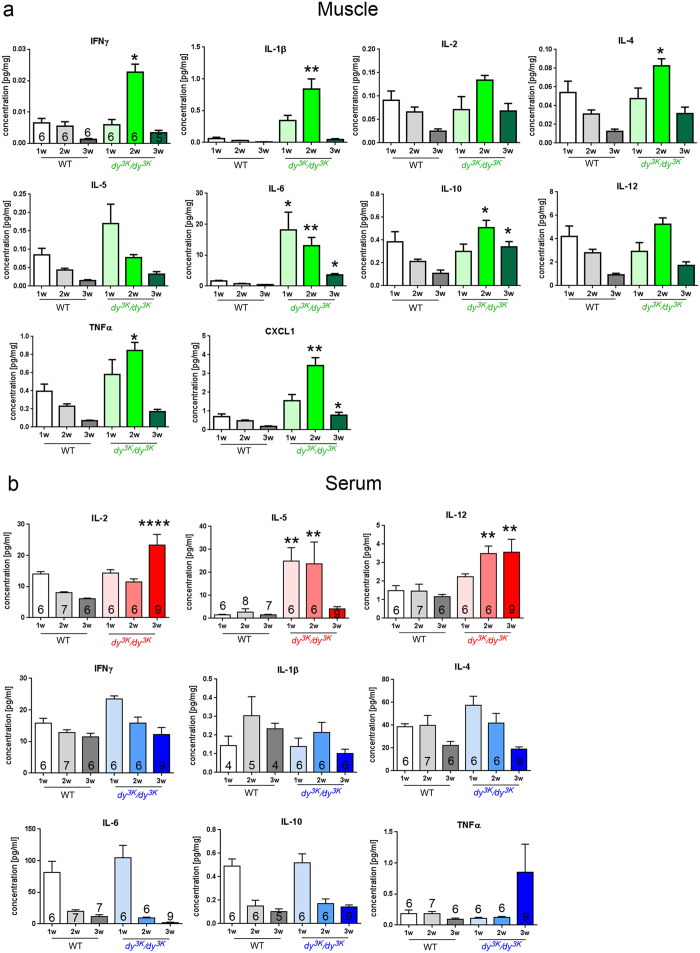
Mesoscale analysis of quadriceps muscle and serum from 1-, 2- and 3-week-old (1w, 2w, 3w) wild-type (WT) and *dy*^*3K*^/*dy*^*3K*^ mice. **(a)** Levels of IFNγ, interleukin-1β, interleukin-4, interleukin-6, interleukin-10, TNFα and CXCL1 are increased in 2-week-old and/or 3-week-old *dy*^*3K*^/*dy*^*3K*^ muscle. Concentrations are presented as pg cytokine/mg protein. The same number of mice as indicated in the first graph was included in all analyses. **(b)** In serum, only interleukin-2, interleukin-5 and interleukin-12 levels are increased in *dy*^*3K*^/*dy*^*3K*^ mice (top row, depicted in red). Non-altered cytokines are presented in blue (middle and bottom rows). Concentrations are presented as pg cytokine/ml serum. The number of mice included at each time point is indicated in the graphs. Stars indicate significant difference between dystrophic and age-matched wild-type mice (Kruskal-Wallis test followed by Dunn’s test).

Since inflammation could potentially dictate the muscle disease outcome, it is urgent to characterize the immunological milieu in laminin α2 chain-deficient muscle. To our knowledge, the cytokine expression profile has not been examined in *dy*^*3K*^/*dy*^*3K*^ mice over a time course of the disease. Consequently, we aimed to assess cytokine levels in early pathology (1-week-old), intermediate disease stage (2-week-old) and late pathology (3-week-old) of *dy*^*3K*^/*dy*^*3K*^ mice in comparison to age-matched wild-type animals. Cytokine analysis revealed common pattern of robust cytokine peak in 2-week-old dystrophic muscle and subsequent drop of cytokine levels, both pro- and anti-inflammatory, at 3 weeks of age (Fig. 1a), suggesting a time-dependent association of the muscle inflammation. Such mechanism of cumulated, intensified inflammatory response with overlap of pro- and anti-inflammatory signals is different from inflammation characteristics in healing normal muscle and resembles the inflammatory status in muscle with impaired regeneration^48^. We did not observe this general pattern when analysing the same cytokines in circulation. Rather, serum levels of interleukin-1β, interleukin-4, interleukin-6, TNFα, interleukin-10 and IFNγ were unaffected by laminin α2-deficiency and only circulating levels of interleukin-2, interleukin-5 and interleukin-12 were increased (Fig. 1b).

**Figure 2:**
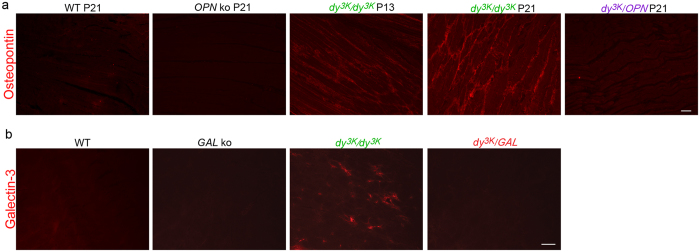
Osteopontin and galectin-3 are upregulated in *dy*^*3K*^/*dy*^*3K*^ muscle. (**a**) Longitudinal hind-limb paraffin sections from 13-day-old (intermediate stage of the disease) and 21-day-old (late pathology) *dy*^*3K*^/*dy*^*3K*^ mice (P13 and P21, respectively); 21-day-old wild-type mice (WT, P21), osteopontin knockout mice (*OPN* ko, P21) and double knockout mice (*dy*^*3K*^/*OPN*, P21) were probed with an anti-osteopontin antibody. Osteopontin is absent from wild-type muscle, but the cytokine expression is highly increased throughout the disease course in *dy*^*3K*^/*dy*^*3K*^ muscle. Multiple muscles from the whole limb were analysed. As expected, osteopontin is not present in osteopontin-deficient muscles (*OPN* ko and *dy*^*3K*^/*OPN*). **(b)** Transverse cryosections of quadriceps muscle from 3-week-old WT, galectin-3 knockout (*GAL* ko), *dy*^*3K*^/*dy*^*3K*^, and double knockout (*dy*^*3K*^/*GAL*) mice stained for galectin-3. Galectin-3 is only expressed in *dy*^*3K*^/*dy*^*3K*^ muscle. Bars: 50 μm.

Both osteopontin and galectin-3 have been shown to influence and/or to be influenced by the numerous cytokines studied above. For example, IFNγ, interleukin-1β, TNFα and interleukin-10 are involved in osteopontin and galectin-3 regulation in different tissues^49^,^50^,^51^,^52^,^53^,^54^,^55^,^56^. Furthermore, these cytokines contribute either to muscle repair and/or to the pathology of muscular dystrophy^11^,^57^,^58^,^59^,^60^,^61^,^62^. Additionally, augmented expression of osteopontin has been shown to promote disease progression and fibrosis in the *mdx* mouse by stimulation of pro-fibrotic macrophage population^34^,^37^. Finally, osteopontin and galectin-3 are massively upregulated in skeletal muscles from MDC1A patients^31^ as well as in the *dy*^*W*^/*dy*^*W*^ mouse model for the disease^38^,^44^. In the light of these results published by others and in the context of our mesoscale data (Fig. 1), we postulated that osteopontin and galectin-3 could also be disease modulators in MDC1A. In order to determine if osteopontin and galectin-3 deletion prevent muscle wasting in mice with MDC1A, we generated laminin α2 chain-deficient animals that lack osteopontin (*dy*^*3K*^/*OPN)* or galectin-3 (*dy*^*3K*^/*GAL*). Immunostaining with antibodies against osteopontin and galectin-3 confirmed dramatically increased levels in *dy*^*3K*^/*dy*^*3K*^ muscle (Fig. 2).

### Deletion of osteopontin or galectin-3 does not improve the overall health of *dy* ^*3K*^/*dy* ^*3K*^ mice

**Figure 3:**
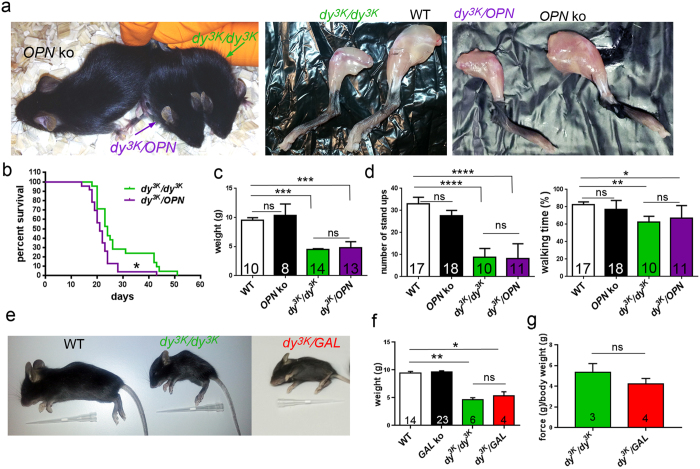
Osteopontin or galectin-3 deletion does not have beneficial influence on general condition of *dy*^*3K*^/*dy*^*3K*^ mice. **(a)** Outward phenotype of 3-week-old single and double knockout mice. Decreased size, emaciation and severe muscle wasting is evident in *dy*^*3K*^/*dy*^*3K*^ and *dy*^*3K*^/*OPN* animals. *OPN* ko littermates are normal. **(b)** *Dy*^*3K*^/*dy*^*3K*^ and *dy*^*3K*^/*OPN* survival curves show decreased life span of animals that lack osteopontin (p = 0.0141, log-rank Cox-Mantel test). N = 21 and n = 23, respectively. **(c)** Body weight analysis of 3-week-old males reveals no significant differences between *dy*^*3K*^/*dy*^*3K*^ and *dy*^*3K*^/*OPN* mice (p > 0.9999). Both are significantly smaller than wild-type and osteopontin knockout littermates (p = 0.0003, p = 0.0009; p = 0.0002, p = 0.0006; respectively, Kruskal-Wallis, followed by Dunn’s test). **(d)** Activity of *dy*^*3K*^/*dy*^*3K*^ and *dy*^*3K*^/*OPN* mice is equally compromised as shown by number of stand ups (left) and walking time measurement (right). *Dy*^*3K*^/*dy*^*3K*^ and *dy*^*3K*^/*OPN* animals were significantly less active than normal littermates, both in the walking test (p = 0.0037 and p = 0.0292, respectively; one-way ANOVA followed by Sidak’s test) and in stand-up test (p < 0.0001, one-way ANOVA followed by Sidak’s test). Both dystrophic mouse strains were equally inactive (p > 0.9999 and p = 0.9193, number of stand ups and walking time, respectively; one-way ANOVA followed by Sidak’s test). **(e)** Double knockout (*dy*^*3K*^/*GAL*) mice are presented with the same outward muscular dystrophy features as single knockout *dy*^*3K*^/*dy*^*3K*^ animals. **(f)** Body weight of wild-type (WT), galectin-3 knockout (*GAL* ko), *dy*^*3K*^/*dy*^*3K*^, and double knockout (*dy*^*3K*^/*GAL*) mice were recorded at 3 weeks of age. Both *dy*^*3K*^/*dy*^*3K*^ and *dy*^*3K*^/*GAL* mice weigh significantly less compared with WT (p = 0.0021 and p = 0.0188, respectively; Kruskal-Wallis test followed by Dunn’s test). **(g)** Removal of galectin-3 does not increase muscle strength in *dy*^*3K*^/*dy*^*3K*^ mice, as demonstrated by grip strength test (forelimbs) (p = 0.3429, Mann-Whitney).

We have analysed the phenotype of all animal genotypes at the age of 1, 2 and 3-weeks, with particular emphasis on week 3 (corresponding to late pathology), when the readout of a mouse condition is the most complete. Neither osteopontin nor galectin-3 single knockout mice (*OPN* ko and *GAL* ko, respectively) display any overt phenotype (Fig. 3 and Supplementary material, Video S1)^15^,^63^. Initial observations of 2-3-week-old *dy*^*3K*^/*dy*^*3K*^and both double knockout mice strains revealed muscular dystrophy symptoms, such as: decreased size, waggling gait, reduced eagerness to move, tremor and hind-limb lameness, muscle wasting and gradual worsening of general health (Fig. 3a,e and Supplementary material, Video S2 and S3). Detailed analysis of the outward phenotype of 3-week-old animals confirmed no improvement of the overall condition of *dy*^*3K*^/*dy*^*3K*^ mice upon osteopontin or galectin-3 deletion. Both *dy*^*3K*^/*OPN* and *dy*^*3K*^/*GAL* animals showed greatly reduced weight compared with healthy controls, but there was no significant difference in body mass between *dy*^*3K*^/*dy*^*3K*^and *dy*^*3K*^/*OPN* or *dy*^*3K*^/*GAL* mice, respectively (Fig. 3c and f). What is more, *dy*^*3K*^/*OPN* mice had a significantly shorter life span than single knockout *dy*^*3K*^/*dy*^*3K*^ animals (Fig. 3b) (median survival 21 and 24 days, respectively). In conclusion, deletion of osteopontin or galectin-3 was not at all beneficial for the general condition of *dy*^*3K*^/*dy*^*3K*^mice.

### Muscle function is not enhanced upon osteopontin or galectin-3 deletion in *dy* ^*3K*^/*dy* ^*3K*^ mice

To compare muscle function in *dy*^*3K*^/*dy*^*3K*^ single knockout and *dy*^*3K*^/*OPN* double knockout mice, we carried out two activity tests on 3-week-old animals (including wild-type and osteopontin*-*null mice): vertical activity test (number of stand ups during 5 minutes) and horizontal activity test (5 minutes locomotion test when exploring a new cage) (Fig. 3d). We found that *dy*^*3K*^/*dy*^*3K*^ and *dy*^*3K*^/*OPN* animals were significantly less active than wild-type mice (Fig. 3d, left and right panel, respectively). Moreover, double knockout mice did not show any improvement of motor activity compared to *dy^3K^/dy^3K^* individuals (Fig. 3d). Even if both *dy*^*3K*^/*dy*^*3K*^ and *dy*^*3K*^/*OPN* animals were to some extent able to explore the new cage (amount of time spent moving), the quality of movement clearly pointed towards impaired muscle function: mice were moving slowly, waddling or wobbling and often tripping (Supplementary Video 2 and 3). To assess muscle strength in *dy*^*3K*^/*dy*^*3K*^ and *dy*^*3K*^/*GAL* mice we used a grip strength meter. Calculation of normalized strength did not reveal any significant difference between *dy*^*3K*^/*dy*^*3K*^ and *dy*^*3K*^/*GAL* mice (Fig. 3g). Thus, muscle function is not improved upon osteopontin or galectin-3 deletion in laminin α2 chain-deficient mice.

### Muscle phenotype is exacerbated upon osteopontin deletion in 2-week-old *dy* ^*3K*^/*dy* ^*3K*^ mice

**Figure 4:**
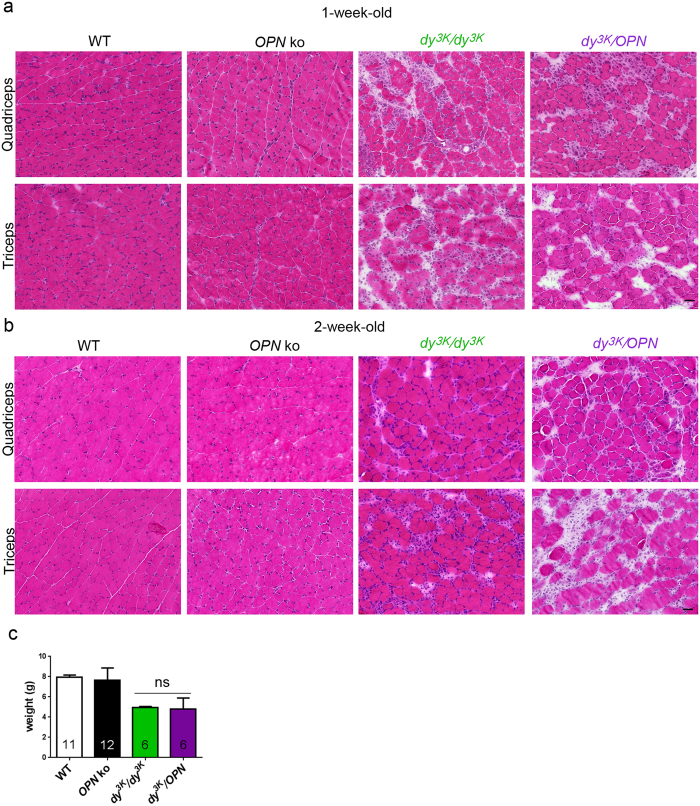
Muscles from 1-week-old and 2-week-old *dy*^*3K*^/*OPN* animals display pronounced muscle damage. **(a)** Hematoxylin and eosin staining of 1-week-old *dy*^*3K*^/*dy*^*3K*^ and *dy*^*3K*^/*OPN* transverse cryosections shows extensive infiltrates of mononuclear cells and disruption of muscle fascicle integrity. **(b)** Muscles from 2-week-old double knockout mice tend to be more affected than muscles from age-matched *dy*^*3K*^/*dy*^*3K*^ mice. Muscle damage and inflammation is intensified in both genotypes at this stage. Osteopontin knockout mice show normal muscles. Five mice from each genotype where analysed. **(c)** There is no difference in body weight between 2-week-old *dy*^*3K*^/*dy*^*3K*^ and *dy*^*3K*^/*OPN* mice (p = 0.9954, Mann-Whitney). Bars: 40 μm.

Severe muscle wasting and loss of muscle function in laminin α2 chain-deficiency is a consequence of muscle fibre damage, muscle cell death, robust inflammatory response and subsequent build-up of fibrotic tissue^2^. Muscle degeneration, followed by inflammation and tissue regeneration (manifested by a presence of small centrally nucleated muscle fibres) are the disease features that occur earliest and are prominent at week 1 and even more so at week 2 in *dy*^*3K*^/*dy*^*3K*^ mice. Thus, we have analysed muscle morphology in single and double knockout mice at these ages. Severe muscle damage and infiltration of mononuclear cells were detected in 1-week-old muscles from both *dy*^*3K*^/*dy*^*3K*^ and *dy*^*3K*^/*OPN* mice (Fig. 4a). Interestingly, muscles from 2-week-old *dy*^*3K*^/*OPN* mice seemed to be more affected than muscles from *dy*^*3K*^/*dy*^*3K*^ single knockouts (especially triceps) (Fig. 4b). They displayed larger areas of deteriorating fibres that were extensively invaded by inflammatory cells (more detailed inflammation analysis from 2-week-old mice is described a few paragraphs below). These observations pointed out that osteopontin deletion could have a negative influence on muscle condition in laminin α2 chain-deficiency. Yet, the weight of 2-week-old double knockout mice was not decreased compared to single knockout *dy*^*3K*^/*dy*^*3K*^ mice (Fig. 4c).

### Muscles from 3-week-old double knockout animals show similar dystrophic features as muscles from *dy* ^*3K*^/*dy* ^*3K*^ single knockout animals

**Figure 5:**
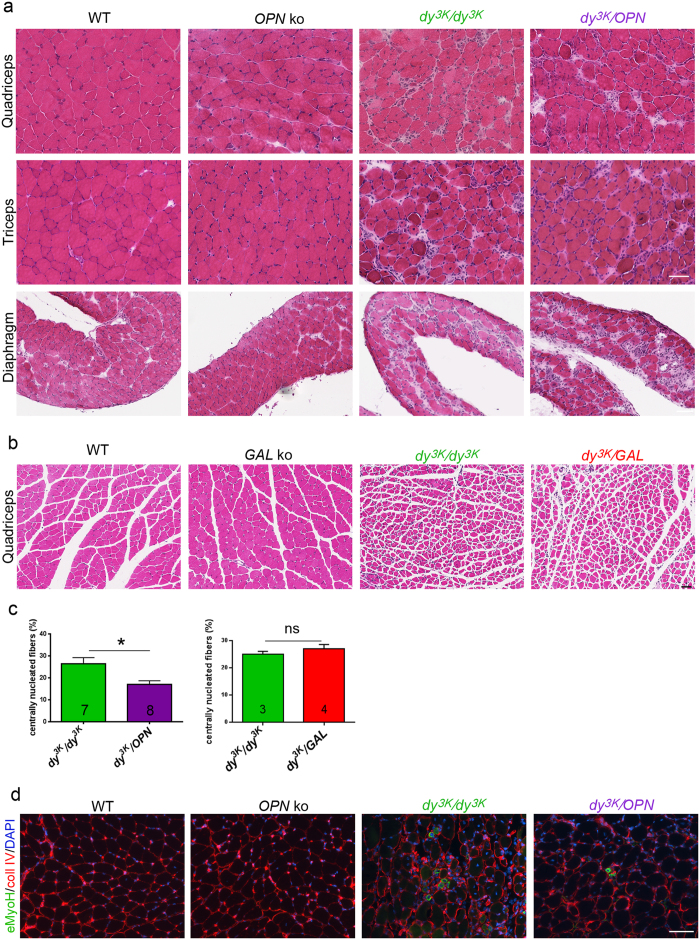
Three-week-old *dy*^*3K*^/*OPN* and *dy*^*3K*^/*GAL* muscles show similar muscular dystrophy hallmarks as *dy*^*3K*^/*dy*^*3K*^ muscles. **(a)** Hematoxylin and eosin staining of quadriceps, triceps and diaphragm cryosections from 3-week-old wild-type (WT), osteopontin knockout (*OPN* ko), *dy*^*3K*^/*dy*^*3K*^ and *dy*^*3K*^/*OPN* animals. Osteopontin*-*null muscles display normal morphology, whereas *dy*^*3K*^/*dy*^*3K*^ and *dy*^*3K*^/*OPN* muscles share dystrophic features of MDC1A: disrupted integrity of muscle fascicles, muscle fibre degeneration/regeneration, infiltration of mononuclear cells, and presence of fibrotic lesions. **(b)** Transverse paraffin sections of quadriceps muscle from 3-week-old wild-type (WT), galectin-3 knockout (*GAL* ko), *dy*^*3K*^/*dy*^*3K*^, and double knockout (*dy*^*3K*^/*GAL*) mice stained with hematoxylin and eosin. Deletion of galectin-3 in *dy*^*3K*^/*dy*^*3K*^ mice does not reduce muscle pathology. Galectin-3 knockout muscle does not display any muscle defects. Bars: 50 μm. **(c)** Osteopontin absence negatively impacts muscle regeneration in laminin α2 chain-deficiency (left panel). The number of regenerating fibres was significantly decreased in triceps from 3-week-old *dy*^*3K*^/*OPN* animals compared with *dy*^*3K*^/*dy*^*3K*^ mice (p = 0.0205, Mann-Whitney). Removal of galectin-3 does not impact muscle regeneration in laminin α2 chain-deficiency (right panel) (p = 0.6286, Mann-Whitney). (**d**) Embryonic myosin heavy chain staining (green) reveals low numbers of newly regenerating fibres in 3-week-old *dy*^*3K*^/*dy*^*3K*^ and *dy*^*3K*^/*OPN* triceps. Collagen IV immunostaining (red) and DAPI labelling (blue) depict muscle fibres and nuclei, respectively. At least five mice from each group were analysed. Bar: 50 μm.

All muscular dystrophy hallmarks (degeneration/regeneration cycles, inflammatory response, fibrosis) were clearly visible in various 3-week-old *dy*^*3K*^/*dy*^*3K*^, *dy*^*3K*^/*OPN* and *dy*^*3K*^/*GAL* muscles, but not in osteopontin or galectin-3 knockout mice (Fig. 5a and b). However, no obvious difference between *dy*^*3K*^/*dy*^*3K*^ and double knockout dystrophic strains was detected at week 3, despite observations that muscle morphology was aggravated in 2-week-old *dy*^*3K*^/*OPN* mice.

Notably, despite its role in muscle pathology progress^34^,^37^, osteopontin has been shown to promote muscle repair^30^,^33^. Consequently, we assessed the impact of osteopontin deletion on muscle regeneration in 3-week-old double knockout animals. Quantification of centrally nucleated fibres (represents overall regeneration) revealed a decrease in regeneration capacity in double knockout muscle compared with *dy*^*3K*^/*dy*^*3K*^ muscle (Fig. 5c). We also analysed whether *dy*^*3K*^/*dy*^*3K*^ and *dy*^*3K*^/*OPN* animals were able to form newly regenerating fibres at 3 weeks of age. Embryonic myosin heavy chain immunostaining revealed very few newly regenerating fibres at the terminal stage of the disease in both dystrophic mouse models, especially in *dy*^*3K*^/*OPN* mice, which showed only spread single positive fibres, whereas *dy*^*3K*^/*dy*^*3K*^ mice showed occasional clusters of regenerating fibres (Fig. 5d). These results further confirm a role for osteopontin in muscle regeneration.

Conversely, deletion of galectin-3 in *dy*^*3K*^/*dy*^*3K*^ mice did not affect myofibre regeneration (Fig. 5c, right graph).

### Fibrotic lesions are not reduced upon osteopontin or galectin-3 deletion from laminin α2 chain-deficient muscle

**Figure 6:**
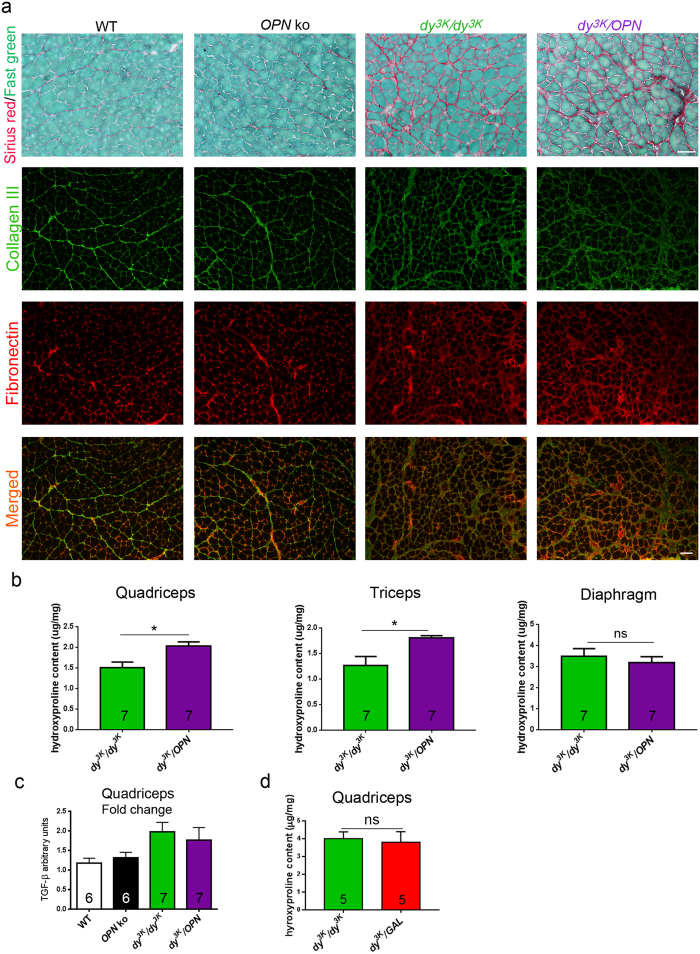
Fibrosis is enhanced in *dy*^*3K*^/*dy*^*3K*^ and *dy*^*3K*^/*OPN* muscles. (**a**) Sirius red staining (visualizes collagens) of 3-week-old quadriceps and immunolabelling for collagen III (green) and fibronectin (red) in 3-week-old triceps reveal no fibrosis prevention upon osteopontin deletion in laminin α2 chain-deficient muscular dystrophy. Collagen III and fibronectin are clearly upregulated in *dy*^*3K*^/*dy*^*3K*^ and *dy*^*3K*^/*OPN* muscles compared with wild-type and osteopontin-null muscles. Bars: 50 μm. **(b)** Quantification of hydroxyproline content in quadriceps, triceps and diaphragm muscle from *dy*^*3K*^/*dy*^*3K*^ and *dy*^*3K*^/*OPN* animals shows significant increase of collagen deposition in double knockout limb muscles (p = 0.0175 and p = 0.0262, respectively), but not in diaphragm (p = 0.7104, Mann-Whitney). **(c)** qPCR analysis of normal (wild-type, osteopontin knockout) and dystrophic (*dy*^*3K*^/*dy*^*3K*^ and *dy*^*3K*^/*OPN*) quadriceps revealed no impact on TGF-β transcript levels upon osteopontin deletion in laminin α2 chain-deficient muscle (p > 0.9999, Kruskal-Wallis, followed by Dunn’s test). **(d)** Fibrosis is equally severe in *dy*^*3K*^/*dy*^*3K*^ and *dy*^*3K*^/*GAL* muscles. Quantification of hydroxyproline content in quadriceps muscle shows no significant difference between single and double knockout mice (p = 0.5476, Mann-Whitney).

Osteopontin promotes formation of collagen fibrils in injured tissue^15^. Fibrosis inhibition upon osteopontin deletion in *mdx* mice was the major consequence of this genetic manipulation^34^. We investigated whether osteopontin ablation prevents the build-up of connective tissue in *dy*^*3K*^/*dy*^*3K*^ mouse model despite its negative impact on muscle repair. Sirius red staining (that visualizes collagens) and immunostaining towards collagen III and fibronectin showed abundant fibrotic lesions in *dy*^*3K*^/*dy*^*3K*^ and *dy*^*3K*^/*OPN* muscles (Fig. 6a). What is more, quantification of hydroxyproline in quadriceps and triceps showed increased collagen content in *dy*^*3K*^/*OPN* limb muscles compared with *dy*^*3K*^/*dy*^*3K*^ muscles (Fig. 6b). However, such upregulation of collagen was not evident in *dy*^*3K*^/*OPN* diaphragm (Fig. 6b). This data indicates that slightly impaired regeneration in *dy*^*3K*^/*OPN* limb muscle (Fig. 5c) together with increased inflammatory response in 2-week-old mice (Fig. 4b, see also below) possibly result in augmented fibrosis.

Since fibrosis reduction in *mdx/OPN* muscle was associated with decreased TGF-β gene expression, we analysed whether fibrosis enhancement upon osteopontin deletion in laminin α2 chain-deficiency is a consequence of a TGF-β-related mechanism. TGF-β has been shown to be upregulated in MDC1A^13^,^64^,^65^. The analysis of TGF-β gene expression revealed no reduction of TGF-β transcript levels upon osteopontin deletion in *dy*^*3K*^/*dy*^*3K*^ muscle (Fig. 6c). Taken together, loss of osteopontin has an opposite effect on fibrotic tissue build-up in *dy*^*3K*^/*dy*^*3K*^ mice compared to *mdx* mice.

Mounting evidence demonstrates that galectin-3 plays a pro-fibrotic role in organ fibrogenesis and galectin-3 inhibitors can protect against fibrotic disorders^46^,^66^. However, deletion of galectin-3 did not reduce fibrosis in *dy*^*3K*^/*GAL* muscle, as revealed by Sirius red staining (Supplementary Fig. S1) and hydroxyproline assay (Fig. 6d).

### Inflammatory response in *dy* ^*3K*^/*dy* ^*3K*^ dystrophic muscle is moderately influenced by osteopontin ablation

**Figure 7:**
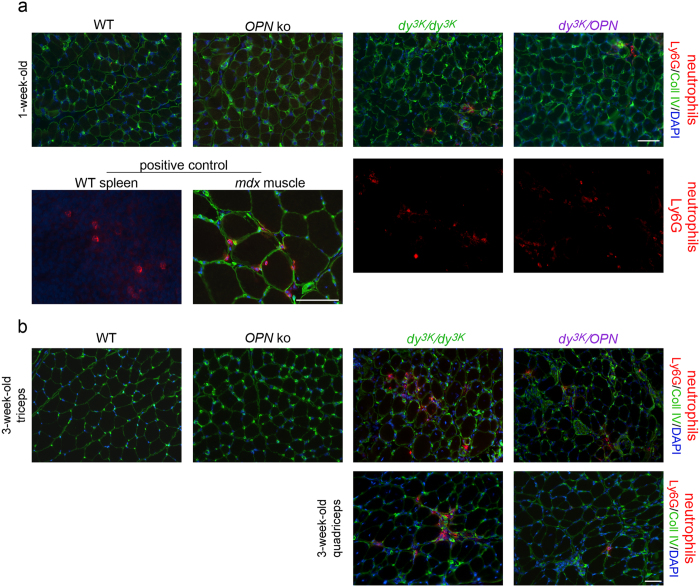
Detailed characterization of neutrophil-mediated immune response in *dy*^*3K*^/*dy*^*3K*^ and *dy*^*3K*^/*OPN* muscles. Osteopontin deletion reduces neutrophil numbers in double knockout muscle. **(a)** One-week-old muscles display the same pattern of neutrophil distribution in *dy*^*3K*^/*dy*^*3K*^ and *dy*^*3K*^/*OPN* mice (Ly6G, red staining). Triceps and quadriceps (cryosections) were analysed and both muscles showed similar characteristics of neutrophil infiltration. Either none or very few neutrophils are present (top panel, triple staining, triceps is shown), or they are occasionally more pronounced focally (bottom panel, single labelling, quadriceps is shown). No neutrophils were detected in wild-type and osteopontin-null muscles. **(b)** Labelling against Ly6G (red) in 3-week-old muscles (triceps and quadriceps) showed almost none or very few neutrophils in double knockout mice. A unique area with relatively many neutrophils in *dy*^*3K*^/*OPN* triceps is presented (top panel), which is still less affected than an average *dy*^*3K*^/*dy*^*3K*^ site. A representative picture of *dy*^*3K*^/*OPN* quadriceps with very few positive cells is shown (bottom panel). Five mice from each group were analysed in (**a**) and (**b**). Sections were co-stained with collagen IV antibody (green) and DAPI (blue) to depict muscle fibres and nuclei, respectively. Ly6G immunolabelling in spleen and *mdx* muscle is shown as positive control. Bars: 50 μm.

Both osteopontin and galectin-3 are potent regulators of inflammatory cells. In particular, they modulate the activity of macrophages and neutrophils^23^,^26^,^37^,^67^,^68^,^69^,^70^, major inflammatory cell types in *mdx* muscle^34^. In MDC1A, macrophages have been suggested to constitute a major population of inflammatory cells^10^,^11^,^71^. We investigated the contribution of neutrophils to inflammation in *dy*^*3K*^/*dy*^*3K*^ muscle. Ly6G staining (depicting neutrophils) was clearly less pronounced than CD11b staining (labelling both macrophages and neutrophils), suggesting that neutrophils are not as numerous as macrophages in 3-week-old *dy*^*3K*^/*dy*^*3K*^ muscle (Supplementary Fig. S2). Since neutrophils were shown to be reduced in *mdx/OPN* mice^34^, we inspected the impact of osteopontin deletion on neutrophils in laminin α2 chain-deficiency. In 1-week-old *dy*^*3K*^/*dy*^*3K*^ and *dy*^*3K*^/*OPN* mice, at the onset of inflammation, muscles were mildly invaded by neutrophils (Fig. 7a, top panel). However, occasional large clusters of neutrophils were present in some (but not all) analysed muscles among both *dy*^*3K*^/*dy*^*3K*^ and *dy*^*3K*^/*OPN* individuals (Fig. 7a, bottom panel). Consistent with data shown in Supplementary Fig. S2, some neutrophils were observed in *dy*^*3K*^/*dy*^*3K*^ muscle as the disease progressed (3-week-old mice) (Fig. 7b). Almost no neutrophils were detected in 3-week-old *dy*^*3K*^/*OPN* muscles (Fig. 7b), suggesting that osteopontin deletion influences neutrophil population in *dy*^*3K*^/*dy*^*3K*^ muscle. This data is in line with findings in *mdx* mice deficient in osteopontin^34^.

**Figure 8:**
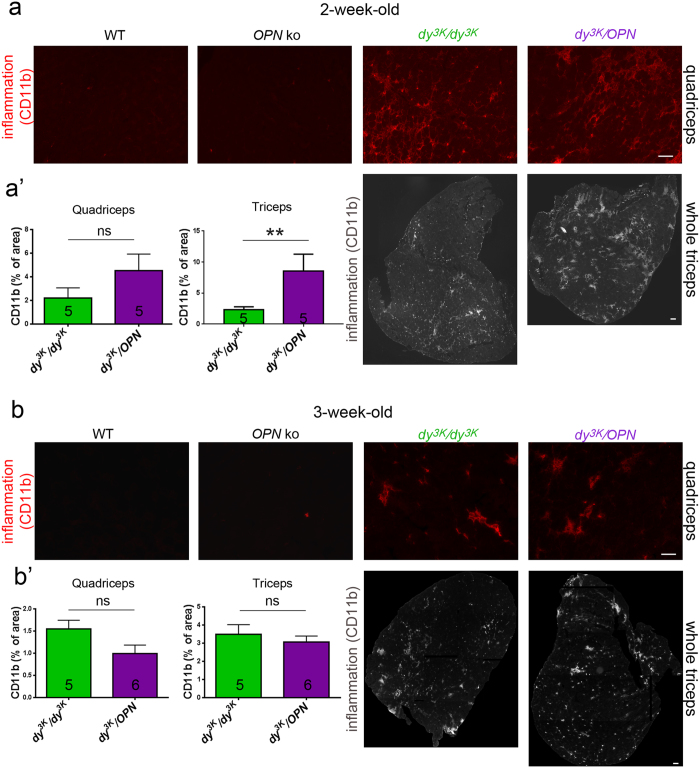
The intensity of inflammatory infiltration varies between *dy*^*3K*^/*dy*^*3K*^ and *dy*^*3K*^/*OPN* muscles at different stages of the disease. **(a)** Muscles from 2-week-old *dy*^*3K*^/*OPN* mice are more inflamed (especially triceps, bottom panel), as demonstrated by immunostaining against CD11b. **(a’)** Quantification of CD11b-stained muscle area in quadriceps and triceps confirms a strong trend for increased inflammation in limb muscles from double knockout mice (p = 0,0952 and p = 0.0079, for quadriceps and triceps, respectively; Mann-Whitney). **(b)** Inflammation is sustained in 3-week-old *dy*^*3K*^/*dy*^*3K*^ and *dy*^*3K*^/*OPN* mice, to the same degree in both dystrophic groups. **(b’)** Quantification of CD11b immunolabelling in both quadriceps and triceps reveals no significant difference between 3-week-old *dy*^*3K*^/*dy*^*3K*^ single knockout and double knockout mice (p = 0.0952 and p = 0.9004, respectively; Mann-Whitney). For both (**a**) and (**b**) a representative area of CD11b-stained quadriceps (red staining, top panel) and a whole CD11b-labelled triceps are shown (white staining, bottom panel). No inflammation was detected in wild-type and osteopontin-null muscles. Five mice from each group were analysed. Bars: 50 μm and 100 μm.

We next analysed the degree of general inflammatory response in muscle (staining against CD11b: marker for monocytes, macrophages, granulocytes and natural killer cells) from 1, 2 and 3-week-old mice (Supplementary Fig. S3, Fig. 8 and Supplementary Fig. S4). Inflammatory cells invade the laminin α2 chain-deficient muscle already at the age of 1 week^10^ (Supplementary Fig. S3a), increase in numbers at 2 weeks of age (Fig. 8a) and remain pronounced at the terminal stage of the disease (week 3) (Fig. 8b). Infiltrates of CD11b positive inflammatory cells were also clearly evident in 1-week-old, 2-week-old and 3-week-old *dy*^*3K*^/*OPN* muscles, as well as in 3-week-old *dy*^*3K*^/*GAL* muscles (Supplementary Fig. S3a, Fig. 8 and Supplementary Fig. S4). We subsequently quantified the area covered by inflammatory cell infiltrates at those stages of the disease in *dy*^*3K*^/*dy*^*3K*^ and *dy*^*3K*^/*OPN* muscles. No significant difference between single and double knockout animals was found for either 1-week-old triceps (Supplementary Fig. S3a’) or 3-week-old quadriceps and triceps (Fig. 8b’). However, as concluded from muscle histology analysis (Fig. 4), we detected a transient increase of inflammatory response in 2-week-old *dy*^*3K*^/*OPN* muscles (Fig. 8a’). Similar to osteopontin removal, deletion of galectin-3 in *dy*^*3K*^/*dy*^*3K*^ muscle did not seem to influence the general inflammatory response in 3-week-old animals (Supplementary Fig. S4).

**Figure 9:**
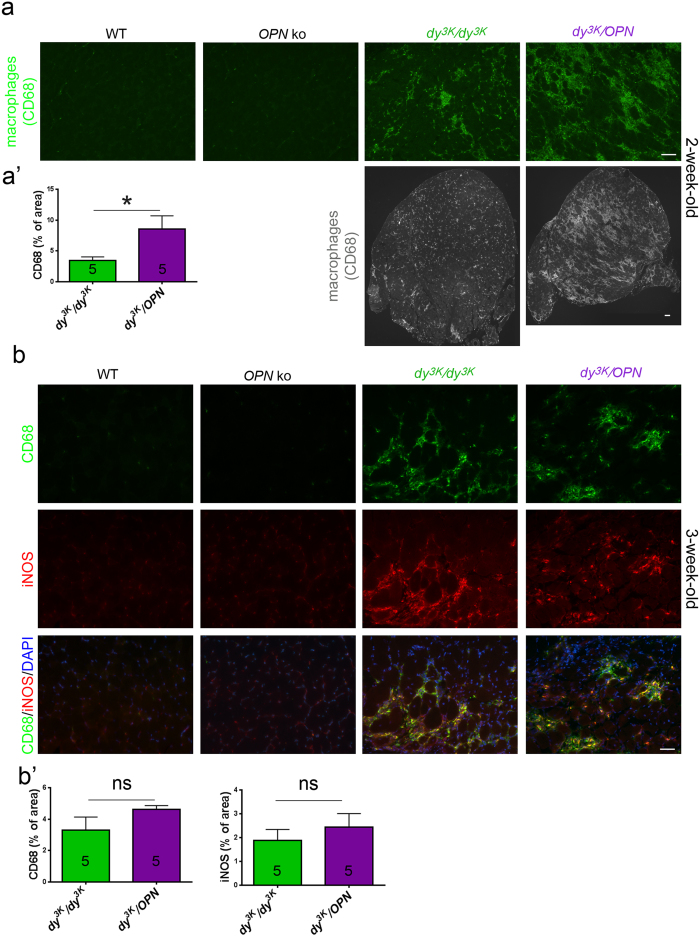
Osteopontin deletion in laminin α2 chain-deficient muscle temporarily influences macrophage numbers, but does not change the general character of macrophage-mediated immune response. **(a)** CD68 immunostaining of 2-week-old *dy*^*3K*^/*dy*^*3K*^ and *dy*^*3K*^/*OPN* triceps muscle reveals increased infiltration of macrophages/monocytes in double knockout muscle. Representative areas of triceps and whole triceps are shown. **(a’)** Quantification of CD68-stained muscle area confirms significant escalation of macrophage numbers in 2-week-old double knockout mice compared to *dy*^*3K*^/*dy*^*3K*^ single knockout mice (p = 0.0317, Mann Whitney) **(b)** Increase in macrophage numbers in younger double knockout mice is only transient. Three-week-old triceps muscle from both *dy*^*3K*^/*dy*^*3K*^ and *dy*^*3K*^/*OPN* mice show the same appearance when stained against CD68 (top panel, green staining). M1 macrophages are present in both *dy*^*3K*^/*dy*^*3K*^ and *dy*^*3K*^/*OPN* triceps, as demonstrated by immunostaining against iNOS (red, middle panel) and CD68 (merged staining, bottom panel). iNOS is expressed at low levels in vessels in wild-type and osteopontin-deficient muscle. **(b’)** Quantification of CD68 and iNOS labelling confirms no change of M1 macrophage-mediated immune response upon osteopontin deletion in 3-week-old mice (p = 0.2222 and p = 0.6905, respectively; Mann-Whitney). Five mice from each group were analysed in (**a**) and (**b**). Bars: 50 and 100 μm.

Although the numbers of macrophages were not affected upon osteopontin deletion in the *mdx* mouse model^34^, macrophage population was shifted toward pro-regenerative phenotype^37^. Thus, we have studied whether osteopontin removal has the same impact on macrophages in MDC1A (in different inflammatory milieu). We have in fact detected increased numbers of monocytes/macrophages in 2-week-old *dy*^*3K*^/*OPN* muscle compared to *dy*^*3K*^/*dy*^*3K*^ muscle (CD68 immunostaining) (Fig. 9a and a’). In agreement with CD11b staining, this difference in macrophage numbers was not maintained in 3-week-old double knockout mice (Fig. 9b and b’, left graph).

Since M1 macrophages were reduced in *mdx/OPN* mice, we further analysed whether osteopontin deletion influenced the M1 macrophage population. For that purpose we stained 3-week-old muscle sections for iNOS, which is highly produced by M1 pro-inflammatory macrophages in response to an inflammatory stimulus. Since iNOS can also be expressed by endothelial cells and vascular smooth muscle cells, we used CD68 antibody to depict macrophages that produce iNOS. CD68/iNOS positive cells were abundant both in *dy*^*3K*^/*dy*^*3K*^ and *dy*^*3K*^/*OPN* muscles (Fig. 9b), showing no depletion of M1 macrophages in double knockout muscles. This was further confirmed by quantification of iNOS staining (Fig. 9b’, right graph).

These data indicate that deletion of galectin-3 or osteopontin from laminin α2 chain-deficient muscle does not, or only mildly influence the general character of inflammatory response to tissue damage. Additionally, inflammation analyses from *dy*^*3K*^/*dy*^*3K*^ muscle lacking osteopontin only partially match the results obtained from *mdx* mice deficient in osteopontin^34^,^37^.

### Osteopontin deletion does not improve the phenotype of milder mouse model for laminin α2 chain-deficiency (*dy* ^*2J*^/*dy* ^*2J*^)

We also investigated the condition of *dy*^*2J*^/*OPN* mice (*dy*^*2J*^/*dy*^*2J*^animals express low levels of truncated laminin α2 chain) to exclude the possibility that the general severity of the *dy*^*3K*^/*dy*^*3K*^ phenotype is the cause of no health improvement upon osteopontin ablation. Analysis of muscle histology and immunostainings against collagen III and CD11b (macrophages) revealed no differences between *dy*^*2J*^/*dy*^*2J*^ and *dy*^*2J*^/*OPN* muscle phenotype (Supplementary Fig. S5). Additionally, *dy^2J^/OPN* mice showed equal impairment of force generation as *dy^2J^/dy^2J^* littermates in grip strength testing (Supplementary Fig. S5, graph).

## Discussion

The scientific community still struggles to find cure for muscular dystrophy, although great strides towards understanding of the disease pathogenesis have been made. Since genetic interventions that aim at restoration of a mutated gene have encountered numerous obstacles, prevention of secondary defects that accompany a protein loss has become an attractive target for therapy of muscular dystrophy. Development of anti-apoptotic, anti-fibrotic, anti-atrophic, and pro-regenerative treatments has been extensively explored^2^,^72^,^73^,^74^,^75^,^76^. Additionally, anti-inflammatory approaches have also yielded attention^34^,^61^,^75^,^76^,^77^, as inflammation could be a potent modulator of the disease outcome. Yet, we only begin to understand molecular interactions in inflammatory milieu of dystrophic muscle.

In the present study, we examined the role of two pro-inflammatory and pro-fibrotic molecules, osteopontin and galectin-3, in MDC1A. Osteopontin or galectin-3 deletion in *dy*^*3K*^/*dy*^*3K*^ mouse model for MDC1A did not positively influence the mouse phenotype at any time point of the disease course. Quite in contrary, removal of osteopontin even worsened the condition of *dy*^*3K*^/*dy*^*3K*^ animals. These results differ from data obtained from *mdx* mice that lack osteopontin, although similar level of osteopontin up-regulation was detected in both dystrophin- and laminin α2 chain-deficient mouse models^34^,^38^. There might be a few reasons why *dy*^*3K*^/*dy*^*3K*^ mice responded to this genetic manipulation in a diverse manner. First, inflammatory milieu in various types of muscular dystrophy is unique: different subset of inflammatory cells and molecules involved may create a distinctive molecular signature that steers the disease progress. In muscle from Duchenne muscular dystrophy patients and from *mdx* mice a chronic inflammatory state is present^78^,^79^,^80^. The nature of inflammation in MDC1A muscles is somewhat different. The inflammatory response both in patients and in *dy*^*3K*^/*dy*^*3K*^ mice takes place at the early stage of disease, being one of the first features of muscle pathology^1^,^9^,^10^,^11^,^81^. However, the inflammatory response in patients is only transient^1^. Yet, detailed characterization of inflammation in MDC1A is still missing. In particular, extending our knowledge about macrophage populations and behaviour in MDC1A could be vital, since they appear to be significant for the condition of dystrophic muscle (at least in dystrophin-deficiency)^37^,^58^. Our study shows that osteopontin deletion in laminin α2 chain-deficiency did not reduce the population of M1 macrophages and thus did not contribute to change in proportions between proinflammatory/proregenerative macrophages - a shift that improved the phenotype of the *mdx/OPN* mouse^37^. This could explain why ablation of the osteopontin gene in *dy*^*3K*^/*dy*^*3K*^ mice did not result in a similar amelioration of the phenotype. The observed decrease of neutrophil numbers upon osteopontin deletion was clearly not sufficient to reverse the pathology of *dy*^*3K*^/*dy*^*3K*^ mice. Additionally, a temporary acute increase of inflammatory response in 2-week-old *dy*^*3K*^/*OPN* mice could definitely have a tremendous impact on phenotype worsening in older mice (week 3).

Therefore, it is possible that osteopontin in MDC1A inflammatory milieu is in fact beneficial for muscle condition as well as for overall health, as its absence caused further decline in survival and deterioration of already severe muscle phenotype in *dy*^*3K*^/*dy*^*3K*^ mice. Two crucial osteopontin functions in MDC1A are unravelled by our study: 1) osteopontin is involved in muscle regeneration 2) osteopontin does not play a key role as a pro-fibrotic molecule in laminin α2 chain-deficient skeletal muscle. Contrary, it could have an anti-fibrotic effect in MDC1A. Yet, the mechanism of its action is not entirely clear, although it does not seem to involve TGF-β signalling. Osteopontin plays a major role in several fibrotic diseases in different tissues^19^,^20^,^21^. It promotes formation of collagen fibrils^15^, but most likely other molecules compensated for osteopontin absence in *dy*^*3K*^/*dy*^*3K*^ mice and stimulated collagen production in limb muscles to even higher degree. It might also be that increased inflammation in *dy*^*3K*^/*OPN* muscle at the early stage of the disease contributes to compromised tissue repair and augmented fibrosis. Otherwise, impaired regeneration due to osteopontin absence could be the primary cause of inflammation boost and subsequent build-up of fibrotic lesions. Considering that osteopontin has a multidirectional impact on myoblasts, myofibroblasts and inflammatory cells, a link between osteopontin, these cells and muscle regeneration and fibrosis needs to be established. So far an association between regulatory T cells and muscle repair has been clearly demonstrated^82^ and osteopontin seems to influence regulatory T cell numbers^34^.

Alternatively, we postulate that the severity of laminin α2-chain-deficiency could lie behind the failure of disease amelioration upon osteopontin deletion in two mouse models (*dy^3K^/dy^3K^* mouse in particular). It is clearly in contrast to the *mdx* animals, which show a mild phenotype (milder than moderately affected *dy^2J^/dy^2J^* mice) with a lifespan that is close to normal^83^,^84^. In fact, a large number of the therapeutic approaches in *mdx* mice have been successful, ranging from genetic manipulations that aim at dystrophin restoration, through pharmacological approaches targeting secondary defects of the disease, to even protein therapy^73^,^76^,^85^,^86^. When similar therapeutic approaches are tested in the *dy*^*3K*^/*dy*^*3K*^ mouse or in other mouse models for laminin-deficiency, they commonly fail or result in only a modest improvement of the phenotype^2^,^87^,^88^. Nevertheless, our current results and a wide range of previous studies, strongly suggest that for convincing preclinical analysis the choice of animal models that adequately mirror human condition is vital. Another issue to consider is that we developed constitutive double knockout models. We cannot be entirely certain whether gene inactivation of osteopontin and galectin-3 at specific time points would modulate the *dy*^*3K*^/*dy*^*3K*^ phenotype differently.

In summary, our results emphasize that osteopontin may exert different biological functions in different types of muscular dystrophy. This knowledge needs to be considered when designing new therapeutic interventions for muscular dystrophies that aim at modifying inflammatory molecules. Our data question whether targeting osteopontin should be considered as a valid therapeutic approach for MDC1A.

The role of galectin-3 in muscular dystrophy has never been investigated previously. Similar to osteopontin, galectin-3 is a modulator of inflammation and capable of promoting fibrogenesis in different organs and currently galectin-3 inhibition is tested clinically. We now demonstrate that deletion of galectin-3 does not reduce fibrotic scarring in *dy*^*3K*^/*dy*^*3K*^ muscle, suggesting that during chronic muscle damage, galectin-3 may not be a major pro-fibrotic component. Clearly, the function of galectin-3 is dependent on type of injury and the context of organ damage and it cannot be ruled out that modulating galectin-3 expression in other types of muscular dystrophy could be beneficial. Also, it should be noted that galectin-1 protein therapy in *mdx* mice resulted in reduced muscle damage, partly by stabilizing the muscle cell membrane via upregulation of integrin α7β1 and utrophin^89^. This approach remains to be tested in muscular dystrophy caused by laminin α2 chain-deficiency.

Since the molecular signature of inflammation is evidently complex, modulating inflammation in different types of muscular dystrophy should be carefully designed and probably optimized individually for each patient. Further studies will be essential to verify whether therapies aiming at inhibition of inflammation are beneficial for muscular dystrophy patients. We also possibly need to answer a broader question: how efficient is it to target the secondary defects of muscle disease?

## Materials and Methods

### Animal models

Laminin α2 chain-deficient *dy*^*3K*^/*dy*^*3K*^, *dy*^*2J*^/*dy*^*2J*^ 90,91, osteopontin-null (*OPN* ko) and galectin-3 knockout mice (*GAL* ko) were previously described^4^,^63^,^92^. Double knockout animals (*dy*^*3K*^/*OPN* or *dy*^*3K*^/*GAL*) and respective control mice (*OPN* ko, *GAL* ko and wild-type (WT) were generated by breeding *dy*^*3K*^/+*; OPN*/+ or *dy*^*3K*^/+*; GAL*/+ males and females. All experimental procedures involving animals were approved by the Malmö/Lund (Sweden) Ethical Committee for Animal Research (the ethical permit number: M15-12 and M152-14) in accordance with the guidelines issued by the Swedish Board of Agriculture.

### Activity test

Locomotion features of 3-week-old mice were assessed by measuring the walking time and number of stand-ups within 5 first minutes of presence in a new cage^93^. Animals were tested one by one, each in a new empty cage without food. Time was measured when the mouse was active and timer was paused each time the mouse rested. The number of full stand ups was counted at the same time. Mice had not been trained prior to the test.

### Grip strength

Grip strength measurements (forelimbs) were performed on 3-week-old mice *dy^3K^* strain) or 8-9-week-old animals (*dy^2J^* strain) as described before^94^. Briefly, each mouse was allowed to grasp a horizontal metal bar attached to a grip strength meter (Columbus Instruments, Columbus, OH). Upon a good grip (symmetric with both paws) the mouse was gently pulled away until its grasp broke. The test was repeated 5 times for each mouse and the mean value of each session was calculated for analysis. Body weight was recorded in parallel. Animals were not subjected to any training prior to the experiment. Results are presented as normalized strength (force (g)/body weight (g)).

### Animal handling and tissue collection

One-week-old, 2-week-old and 3-week-old control mice, single knockouts and double knockouts were euthanized by cervical dislocation. *Dy*^*2J*^/*dy*^*2J*^ and *dy*^*2J*^/*OPN* mice were sacrificed at week 8. Quadriceps, triceps and diaphragm muscles were dissected for hydroxyproline assay, RNA extraction or immunohistochemistry (embedded in O.C.T compound or in paraffin). Cross sections (7 μm thick for frozen tissues or 5 μm for paraffin embedded tissues) were cut using cryostat (Microm HM 560, Cellab Nordia AB) and microtome (Microm H355, Cellab), respectively.

### Histology and morphometric analysis

Muscle sections were stained with hematoxylin and eosin^10^ or picrosirius red/fast green^6^. Stained cross-sections were scanned using Aperio’s Scanscope CS2. Centrally nucleated muscle fibres and peripherally nucleated normal muscle cells were counted using ImageJ software version 143u. A whole area of each muscle cross section was considered.

### Immunofluorescence

Cryosections were subjected to immunofluorescence labelling with antibodies against: embryonic myosin heavy chain (mouse monoclonal F1.652, 1:10, Developmental Studies Hybridoma Bank, University of Iowa), CD11b (rat monoclonal M1/70, 1:250, BD Pharmingen), Ly6G (rat monoclonal 1A8, 1:200, BD Pharmingen), CD68 (rat monoclonal FA-11, 1:100, AbD Serotec), iNOS (rabbit polyclonal, 1:100, Abcam), collagen III (goat polyclonal, 1:100, Southern Biotech), fibronectin (rabbit polyclonal, 1:1000, Abcam), collagen IV (rabbit polyclonal, 1:200, Millipore) and galectin-3 (rat monoclonal, 1:100).

Paraffin sections were used for staining with osteopontin antibody (rabbit polyclonal AB10910, 1:50, Millipore). Samples were deparaffinized and treated with protease XXIV (20 μg/ml in PBS, Subtilisin Carlsberg, Sigma) for 20 min at RT prior to blocking and staining.

Primary antibodies were detected with relevant secondary antibodies (Molecular Probes). Images were taken using Openlab 4 software. The area corresponding to CD11b, CD68 and iNOS labelling was quantified in relation to the entire area of muscle cross-sections. Pictures covering the whole muscle section were stitched using Photoshop software. In order to avoid false positive signal, artefacts and non-muscle tissue (if present) were removed from the images for the quantification process. Otherwise no modifications were applied to the images. Photos were captured at the same exposure times. At least 5 mice were analysed for each immunostaining. ImageJ software version 143u was used for quantification.

### Mesoscale

Whole blood (from heart puncture) and quadriceps muscles were collected from 1-week, 2-week and 3-week-old wild-type and *dy*^*3K*^/*dy*^*3K*^ mice. Blood samples were processed according to the manufacturer’s specifications (Microvette CB 300, Sarstedt). Muscles were homogenized in Tris Lysis Buffer with proteinase inhibitor tablet (Pierce) using Tissue Lyser (Qiagen). Protein concentration in muscle samples was assessed using Bradford assay. Cytokine and chemokine concentrations were determined by multiplex technology in quadriceps samples and serum (25 μl/sample) using a MSD Mouse Pro-inflammatory V-Plex Plus Kit (IFNγ, interleukin-1β, interleukin-2, interleukin-4, interleukin-5, interleukin-6, interleukin-10, interleukin-12p70, CXCL1, TNFα; K15012C, Mesoscale Discovery) and a SECTOR Imager 6000 (Mesoscale Discovery, Rockville, MD) plate reader according to the manufacturer’s instructions. Data were analysed using MSD Discovery Workbench software. For the muscle homogenate samples the concentrations were normalized to the different protein concentrations measured in the Bradford assay of each homogenized sample^95^.

### Hydroxyproline assay

Quadriceps, triceps and diaphragm muscles from 3-week-old animals were weighed and incubated overnight in concentrated HCl (6 M) at 95 °C (10 mg tissue per 100 μl HCl). For muscle weights ≤20 mg, 200 μl HCl was used. Hydroxyproline standards were treated in the same way as muscle samples. Twenty-five microliters of hydrolysate was neutralized with 25 μl NaOH (6 M) and incubated with 450 μl Chloramine-T reagent (0.056 M) at RT for 25 minutes, followed by incubation with 500 μl Ehrlich’s reagent at 65 °C for 40 minutes. Samples and standards were transferred to a 96-well plate and absorbance was read at 560 nm^6^. All absorbance values were subtracted with blank (0 μg/ml hydroxyproline). Absorbance (*A*560) of standards was plotted against amount of hydroxyproline (microgram) and a linear regression was performed to determine slope and intercept. Hydroxyproline content in measured samples was calculated by equation: *x* (μg) =(*A*560 − *Y*axis intercept)/slope, followed by muscle weight and acid volume adjustment in order to obtain the hydroxyproline content in muscle wet weight (μg/mg).

### RNA isolation and quantitative RT-PCR analysis

RNA was isolated from 3-week-old muscles using Qiagen RNeasy Plus Universal Kit (Qiagen), following the manufacturer’s specifications. The quality and concentration of RNA were assessed using Agilent 2100 Bioanalyzer (Agilent RNA 6000 Nano Kit). One microgram of muscle RNA was used to synthesize cDNA with High Capacity cDNA Reverse Transcription Kit (Applied Biosystems) according to manufacturer’s protocol. The amplification was performed in a LightCycler 480 Real-Time PCR System (Roche), including no-RT control reactions. TaqMan probes detecting mouse TGF-β and β-actin (reference gene) were used (Applied Biosystems). Comparative CT method was used for relative quantitation.

### Statistical analysis

All statistical analyses were performed with GraphPad Prism software version 6. Averaged data were reported as means ± SEM. When comparing more than two groups, D’Agostino-Pearson normality test was applied prior to choosing between one-way ANOVA or Kruskal-Wallis test. To determine significance between two particular groups, Sidak’s or Dunn’s multiple comparison post-hoc tests were applied post one-way ANOVA and Kruskal-Wallis test, respectively. Adjusted p-value was used for assessing statistical significance. For comparisons between two groups Mann-Whitney test was used and statistical significance was accepted for p < 0.05. For survival analysis log-rank Mantel-Cox test was used.

## Additional Information

**How to cite this article:** Gawlik, K. I. *et al*. Potent pro-inflammatory and pro-fibrotic molecules, osteopontin and galectin-3, are not major disease modulators of laminin α2 chain-deficient muscular dystrophy. *Sci. Rep.* **7**, 44059; doi: 10.1038/srep44059 (2017).

**Publisher's note:** Springer Nature remains neutral with regard to jurisdictional claims in published maps and institutional affiliations.

## Supplementary Material

Supplementary Figures

Supplementary Video 1

Supplementary Video 2

Supplementary Video 3
