# Supplementary material for: Potent pro-inflammatory and pro-fibrotic molecules, osteopontin and galectin-3, are not major disease modulators of laminin α2 chain-deficient muscular dystrophy
Source: Sci Rep. 2017 Mar 10;7:44059. doi: 10.1038/srep44059 (PMC5345027; doi:10.1038/srep44059)
Supplement: Supplementary Figures [file srep44059-s1.pdf]

Potent pro-inflammatory and pro-fibrotic molecules, osteopontin and galectin-3, are not major disease modulators of laminin α2 chain-deficient muscular dystrophy

Kinga I. Gawlik[1]*, Johan Holmberg[1*], Martina Svensson [2], Mikaela Einerborg[1], Bernardo M.S. Oliveira[1], Tomas Deierborg[2] and Madeleine Durbeej[1]

[1]Department of Experimental Medical Science, Muscle Biology Unit, Lund University, Sweden

[2] Department of Experimental Medical Science, Experimental Neuroinflammation Laboratory, Lund University, Sweden

*Corresponding authors and equal contribution

*Corresponding Author:
Kinga I. Gawlik, Johan Holmberg
Muscle Biology Unit
Department of Experimental Medical Science
BMC C12, Lund University
221 84 Lund, Sweden
Tel : 0046 46 2220813
Fax: 0046 46 2220855
Email: kinga.gawlik@med.lu.se
johan_k.holmberg@med.lu.se

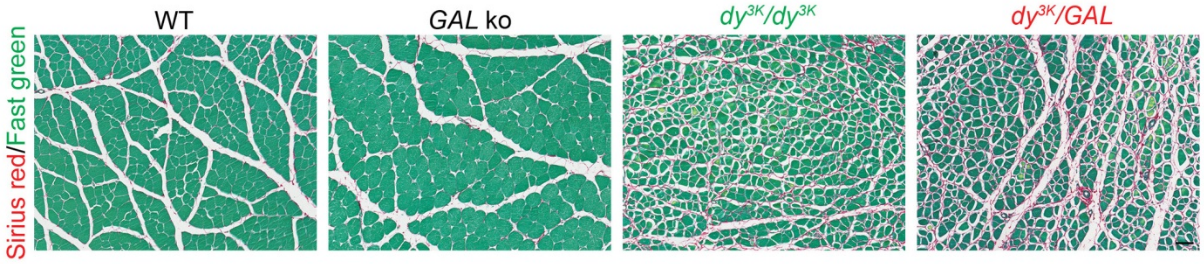

Supplementary Figure 1. Fibrosis is equally severe in *dy*^{3K}/*dy*^{3K} and *dy*^{3K}/*GAL* muscles. Transverse paraffin sections of quadriceps muscle from 3-week-old wild-type (WT), galectin-3 knockout (*GAL* ko), *dy*^{3K}/*dy*^{3K}, and double knockout (*dy*^{3K}/*GAL*) mice stained with Sirius red (visualizes collagen) and Fast green. Fibrotic scarring is pronounced both in *dy*^{3K}/*dy*^{3K} and *dy*^{3K}/*GAL* muscle. Galectin-3 knockout muscle did not display any signs of fibrotic lesions Bar: 50 µm.

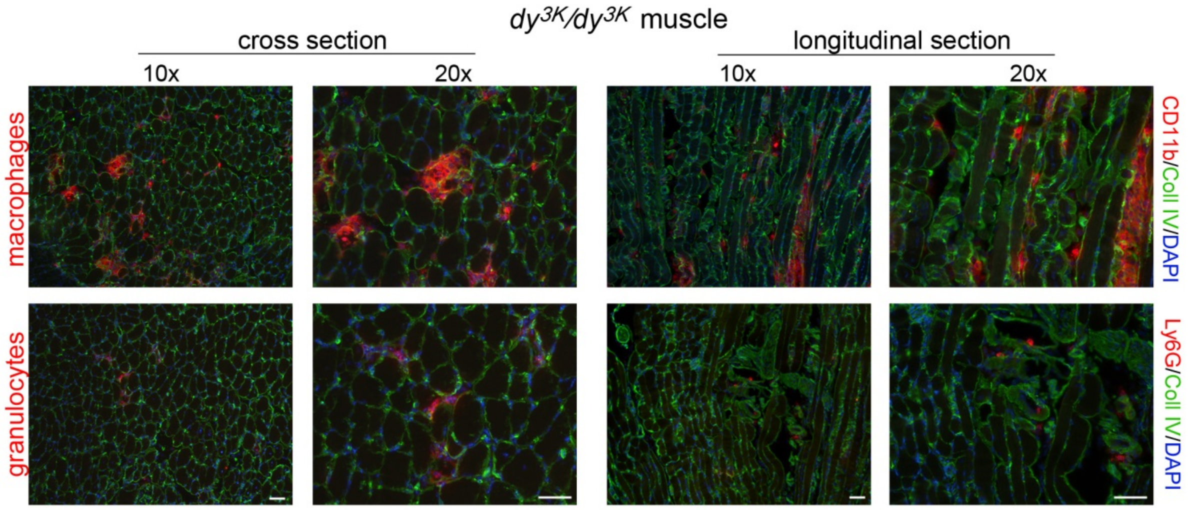

Supplementary Figure 2. Neutrophils are not the major inflammatory cell population in *dy*^{3K}/*dy*^{3K} muscle. Immunolabelling against neutrophils (Ly6G) and leukocytes (CD11b) in 3-week-old *dy*^{3K}/*dy*^{3K} muscle shows moderate signal for neutrophils and strong staining for

broad range of inflammatory cells (red). Corresponding cryosections (quadriceps) for both stainings were chosen to depict the same muscle areas for comparison (for both cross and longitudinal sections). Sections were co-stained with collagen IV (green) and DAPI (blue) to visualize muscle fibers and nuclei, respectively. Three dy^{3K}/dy^{3K} mice were analyzed. Bars: 50 µm.

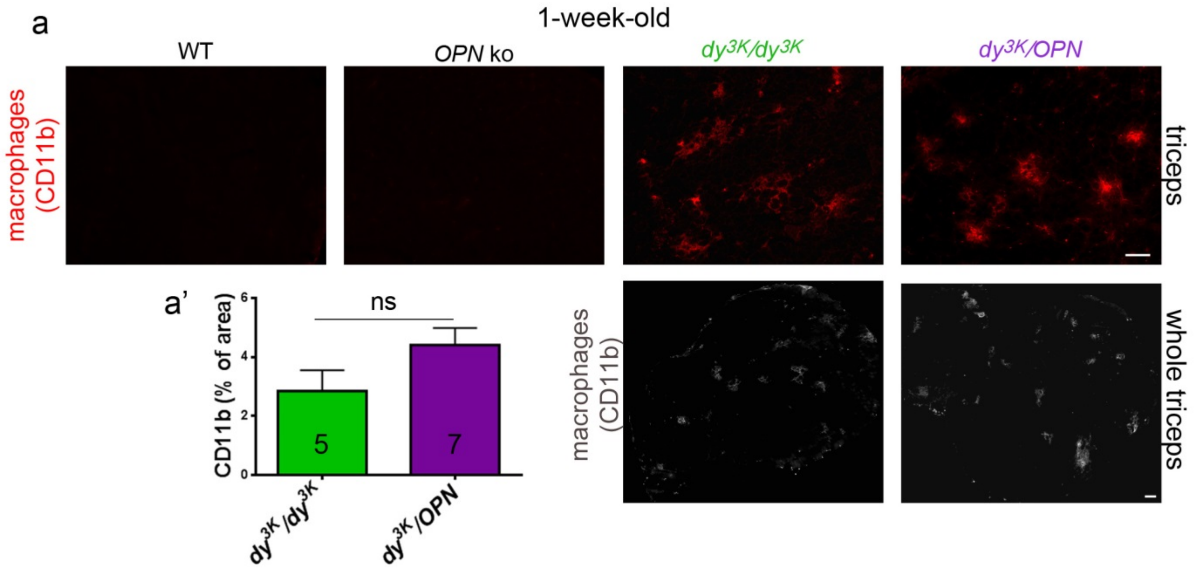

Supplementary Figure 3. (a) Inflammatory cells robustly penetrate already 7-day-old dy^{3K}/dy^{3K} and dy^{3K}/*OPN* muscles (CD11b immunolabelling, cryosections). Severely affected areas of triceps (red staining, top panel) and whole triceps (white staining, bottom panel) are shown. Bars: 50 µm and 100 µm. **(a')** The area corresponding to positive labelling was quantified in triceps from dy^{3K}/dy^{3K} and dy^{3K}/*OPN* mice. Inflammatory cell infiltration did not differ significantly between the two groups ($p=0.1420$, Mann-Whitney).

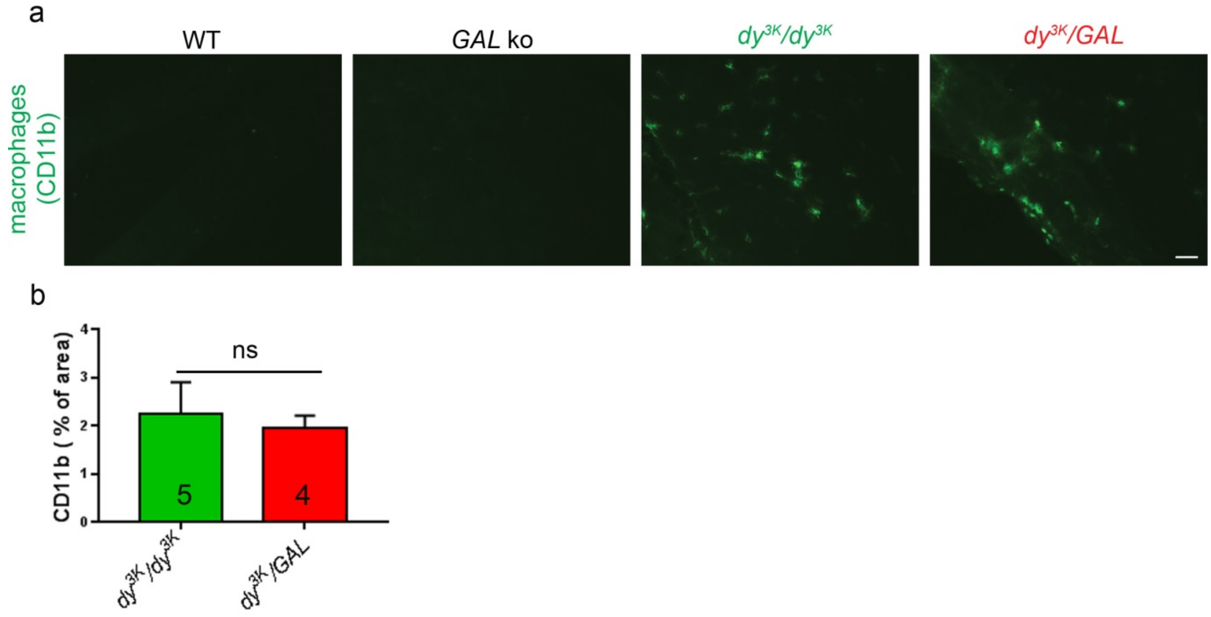

Supplementary Figure 4. The number of inflammatory cells in 3-week-old *dy*^{3K}/*dy*^{3K} muscles is not decreased upon deletion of galectin-3. **(a)** Transverse cryosections of quadriceps muscle from 3-week-old wild-type (WT), galectin-3 knockout (*GAL* ko), *dy*^{3K}/*dy*^{3K}, and double knockout (*dy*^{3K}/*GAL*) mice stained with CD11b. No inflammatory cells were detected in WT and *GAL* ko muscles. Bar: 50 µm. **(b)** The area corresponding to positive labelling was quantified in quadriceps muscle from *dy*^{3K}/*dy*^{3K} and *dy*^{3K}/*GAL* mice. Infiltration of leukocytes did not differ significantly between the two groups (p=0.9048, Mann-Whitney).

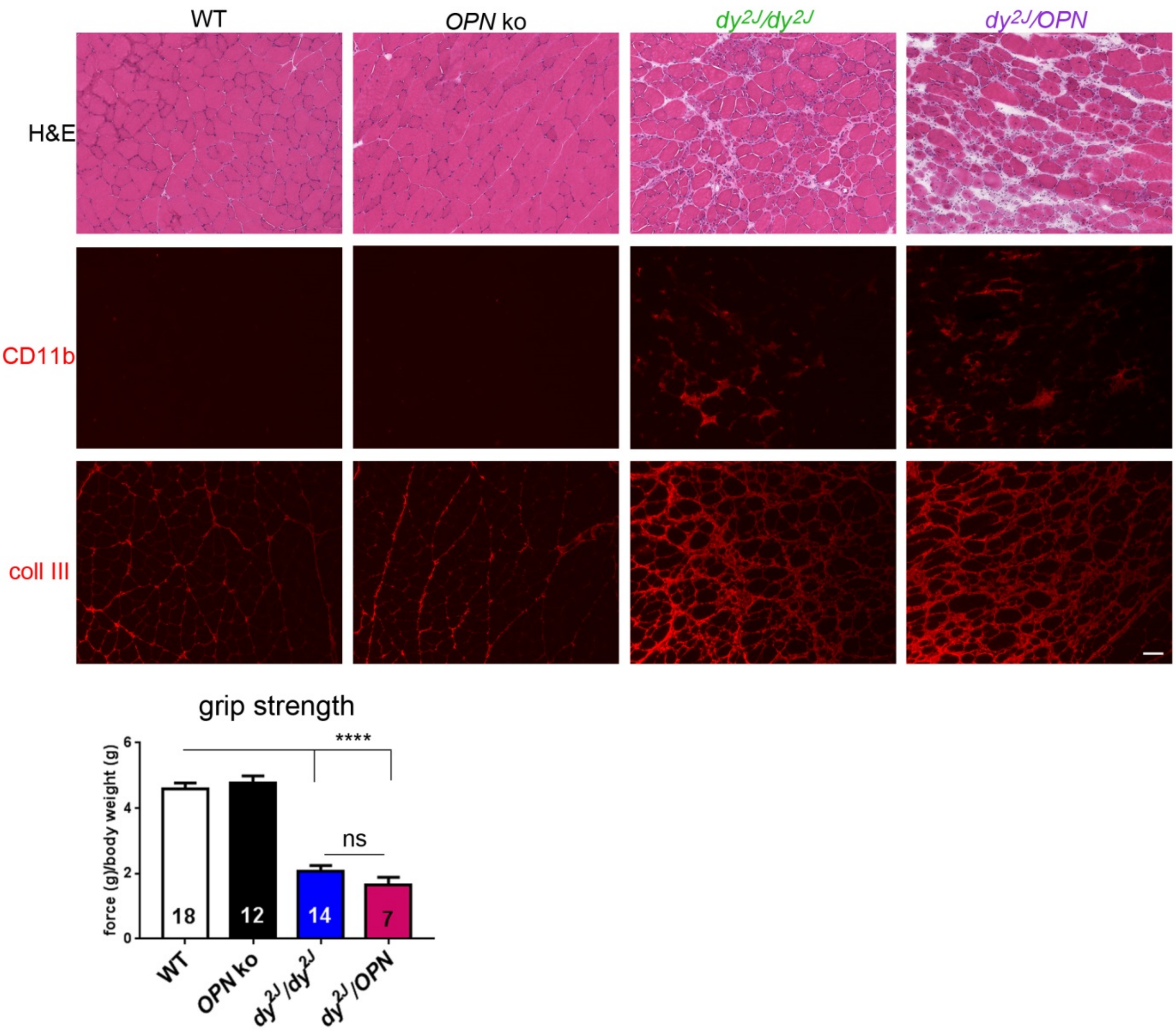

Supplementary Figure 5. *Dy^2J^/OPN* mice are dystrophic. Hematoxylin and eosin staining shows dystrophic features in muscles from both dy^{2J}/dy^{2J} mutant and dy^{2J}/OPN animals (8-week-old). CD11b labelling (red) reveals robust inflammatory response and collagen III immunostaining (red) demonstrates advanced fibrotic scarring in dy^{2J}/dy^{2J} and dy^{2J}/OPN muscles. Bar: 50 µm. Graph: grip strength analysis (forelimbs) reveals no recovery of muscle function in dy^{2J}/OPN mice ($p=0.5920$ vs dy^{2J}/dy^{2J} and $p<0.0001$ vs wild-type; one-way ANOVA followed by Sidak's test).

Video 1, **2 and 3.** *OPN* ko mouse is vivid and active, exploring a new cage very quickly and standing up often (Video 1). Dy^{3K}/dy^{3K} and dy^{3K}/OPN mice are presented with muscular dystrophy features: decreased size, reduced eagerness to move and tremor (Video 2 and 3, respectively). Although they were to some extent able to explore the new cage (amount of time spent moving, see Fig 3d), the quality of movement clearly pointed towards impaired muscle function: mice were moving slowly, waddling or wobbling and often tripping.
